# Deficit of state-dependent risk attitude modulation in gambling disorder

**DOI:** 10.1038/tp.2017.55

**Published:** 2017-04-04

**Authors:** A Fujimoto, K Tsurumi, R Kawada, T Murao, H Takeuchi, T Murai, H Takahashi

**Affiliations:** 1 Department of Psychiatry, Kyoto University Graduate School of Medicine, Kyoto, Japan; 2 Department of Functional Brain Imaging, National Institute of Radiological Sciences, National Institutes for Quantum and Radiological Science and Technology, Chiba, Japan

## Abstract

Gambling disorder (GD) is often considered as a problem of trait-like risk preference. However, the symptoms of GD cannot be fully understood by this trait view. In the present study, we hypothesized that GD patients also had problem with a flexible control of risk attitude (state-dependent strategy optimization), and aimed to investigate the mechanisms underlying abnormal risk-taking of GD. To address this issue, we tested GD patients without comorbidity (GD group: *n*=21) and age-matched healthy control participants (HC group: *n*=29) in a multi-step gambling task, in which participants needed to clear ‘block quota' (required units to clear a block, 1000–7000 units) in 20 choices, and conducted a task-functional magnetic resonance imaging (fMRI) experiment. Behavioral analysis indeed revealed a less flexible risk-attitude change in the GD group; the GD group failed to avoid risky choice in a specific quota range (low-quota condition), in which risky strategy was not optimal to solve the quota. Accordingly, fMRI analysis highlighted diminished functioning of the dorsolateral prefrontal cortex (dlPFC), which has been heavily implicated in cognitive flexibility. To our knowledge, the present study provided the first empirical evidence of a deficit of state-dependent strategy optimization in GD. Focusing on flexible control of risk attitude under quota may contribute to a better understanding of the psychopathology of GDs.

## Introduction

Gambling disorder (GD) is a chronic mental disorder that is characterized by excess gambling in spite of adverse consequences.^1, 2^ GD has been capturing major attention of clinicians and researchers, being the only officially recognized behavioral addiction in the Diagnostic and Statistical Manual of Mental Disorders, fifth edition (DSM-5).^3^ Despite intensive research having been conducted for GD, the underlying mechanisms of abnormal risk-taking remain elusive.^4, 5^

Past studies considered the individual risk preference (trait-like, or ‘static' risk attitude) of GD patients as a fundamental cause of problematic risk-taking. Studies using the Iowa Gambling Task reported that problematic gamblers tended to prefer a risky (high-risk and high-return) deck over a sure (high expected value) deck despite smaller total payoff, demonstrating risk-proneness of GD patients.^6, 7^ Neuroimaging studies showed altered activity of reward-related areas in GD, such as ventral striatum and ventromedial prefrontal cortex, during monetary decision-making tasks, implying neural underpinnings of abnormal risk preference.^8, 9, 10, 11, 12^

Despite accumulated research regarding behavioral/neuronal mechanisms underlying individual risk preference of GD, unsolved riddles remain. For instance, the GD population has a high comorbid rate of mood disorders and anxiety disorders, contrary to the public image of optimistic gamblers.^13, 14^ Moreover, trait risk attitude was not predictable of the risk of relapse.^15^

Recent neuroscience studies concerning risk foraging theory suggested that risk attitude is a rather flexible parameter that reflects a contextual state.^16, 17, 18, 19, 20, 21^ In our previous study, by manipulating the quota severity (required achievements) in a multi-step gambling task, we observed that healthy participants chose safe strategy in lower quota condition, while they chose risky strategy in higher quota condition, demonstrating strategic utilization of risk attitude (state-dependent strategy optimization) in humans.^21^ Accordingly, functional magnetic resonance imaging (fMRI) analysis highlighted a crucial role of the dorsolateral prefrontal cortex (dlPFC), which is responsible for cognitive flexibility,^22, 23, 24^ for encoding of quota severity and exertion of state-dependent strategy optimization.^21^ State-dependent strategy optimization is crucial for adaptive decision-making to achieve a multi-step goal,^25, 26^ and the failure of flexible control of risk attitude may result in unnecessary gambling in GD. To our knowledge, no study has tested this possibility in a GD population.

Here, we aimed to examine whether GD patients have a deficit of state-dependent strategy optimization during decision-making under quota constraint. To this end, we conducted a task-fMRI experiment in GD patients with a multi-step gambling task (Goal-Instructed Gambling task),^21^ and investigated alterations of quota-dependent risk attitude and corresponding neural patterns. We analyzed not only the neural activation pattern, but also its functional connectivity pattern, as impairment of the neural network has been implicated in addiction.^27, 28^ Because the dlPFC played a central role in performing state-dependent strategy optimization, we focused on dlPFC function and functional connectivity pattern in the dlPFC of GD patients. We predicted that GD patients frequently engage in risky choices when they should avoid risk (that is, fail to avoid risk in lower quota condition) due to a failure of state-dependent strategy optimization, and dlPFC activity may reflect such inflexibility during decision-making.

## Materials and methods

### Participants

Twenty-four male GD patients who met the criteria for pathological gambling in DSM-IV-TR and for GD in DSM-V were recruited for the present study. Patients were given the Structured Clinical Interview for DSM-IV (SCID) prior to the experimental sessions, and 3 GD patients were excluded from further analysis because of medication histories for psychiatric disorders (1 for schizophrenia, 1 for alcohol addiction and 1 for attention deficit hyperactivity disorder). Finally, 21 GD patients without comorbidity were analyzed (GD group, *n*=21). The data from age-, sex- and IQ-matched healthy participants analyzed in the previous study were used to configure the healthy control group (HC group, *n*=29). The HC group and the GD group were examined in the same period by the same methods. The sample size was set to exceed 20 participants per group in order to yield statistical power of 0.80 to effect size ~0.90 for between-group analysis.^29^ Of the GD group, 16 patients with maintained abstinence were recruited from a treatment facility, and the remaining 5 patients were recruited from the local community (active gamblers). All participants provided written informed consent before the experimental session. This study was approved by the Committee on Medical Ethics of Kyoto University and was carried out in accordance with the Code of Ethics of the World Medical Association. For further information regarding participants, see Supplementary Information.

### Task and procedure

**Figure 1:**
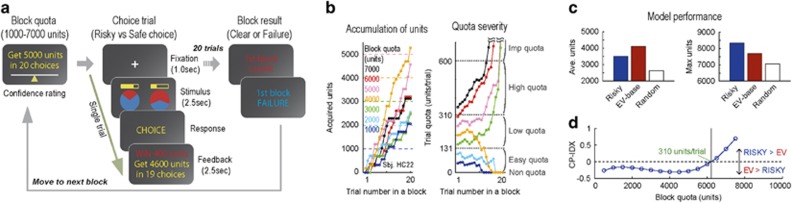
Task and condition-defining simulation. (**a**) Goal-Instructed Gambling Task. A block is begun with block-quota instructions (left row). Participants choose between a risky option and a safe option, and receive a feedback indicating trial quota (units per trial; middle row). Block result is announced after 20 choice trials, and the next block is initiated (right row). (**b**) An example session sequence performed by one subject (subject #22 from HC group). Accumulation of units in a block (left). Color differences indicate block quotas. (right) Transition of trial quota. Dotted lines indicate boundary of five quota conditions. (**c**) Simulation results. Average units earned in 10 000 blocks by computer agents (RISKY model, EV model, random choice; left). Maximum units earned in the simulation (right). (**d**) Transition of relative dominance of RISKY model over EV model computed as CP-IDX. Negative CP-IDX indicates dominance of EV model, and positive CP-IDX indicates dominance of RISKY model. Indifferent point (CP-IDX=0; trail quota=310 unit per trial) indicates boundary of low/high-quota conditions, where optimal strategy is reversed. EV, expected value; HC, healthy controls.

A multi-step gambling task, which was also used in a previous study (Goal-Instructed Gambling Task, Figure 1a),^21^ was adopted for the GD group with identical settings. In this task, participants were required to earn units by making successive gamble choices in order to solve a ‘block quota' in each block (1000–7000 units in 20 choices). The transition of trial quota (remaining units divided by remaining choices, units per trial) was announced as feedback following each choice, and the block result (clear or failure) was announced after the 20th choice in each block. Participants were each paid the same amount (6000 JPY) for their participation, and they were instructed to solve as many blocks as possible. The payment amount was not adjusted according to earning units outcome, ensuring that participants had equal motivation for each block. Participants were also instructed about the maximum payoff of a trial (600 units) before the task session. The program of the behavioral task was constructed and presented using E-prime 2.0 (Psychology Software Tools, Sharpsburg, PA, USA) on a laptop computer (Sony, Tokyo, Japan). In addition to the behavioral session, participants completed self-reported questionnaires, Gambling Craving Scale (GACS)^30^ and BIS/BAS scale,^31^ for measures of subjective craving level and trait impulsivity, respectively.

### Behavioral data analyses

For behavioral analyses, choice trials were classified into five ‘quota conditions': Non-quota condition (0 units per trial), Easy-quota condition (0–131 units per trial), Low-quota condition (131–310 units per trial), High-quota condition (310–600 units per trial), and Imp-quota (impossible quota) condition (>600 units per trial). The boundaries of quota conditions were defined based on the prior computational simulation performed in the previous study^21^ (also see Supplementary Information). Crucially, only low- and high-quota conditions entailed optimal strategy (low: expected value (EV)-based choice, High: risky choice), hence requiring state-dependent strategy optimization.

The risky choice was defined as the choice of a risky option (high magnitude and low winning probability) in a pair of choice options. The high EV choice was defined as the choice of higher EV option in an option pair, in which either risky or safe option could have higher EV. We used corrected threshold *α*=0.01 for correlation analyses between risky choice probability and abstinence period.

### fMRI data analyses

fMRI data obtained during the behavioral session were analyzed. In activation analysis, encoding of quota severity (‘quota severity' contrast, [0 −2 −1 1 2]) was compared between GD and HC groups by two-sample *t*-test (*P*<0.05, cluster-size corrected). In functional connectivity analysis, encoding of state-dependent strategy optimization (‘strategy optimization' contrast, [−1 0 1 1 −1]) was compared between GD and HC groups by two-sample *t*-test (*P*<0.05, cluster-size corrected). To generate psychophysiological interaction (PPI) regressors, the generalized form of the context-dependent PPI (gPPI) method^32^ was employed with left dlPFC seed (peak: [−26, 40, 44]). We confirmed similarity of variance between groups by two-sample F-test for equal variances (*P*>0.10). For further information regarding fMRI data acquisition, activation analysis and functional connectivity analysis, see Supplementary Information.

## Results

### Quota-dependent modulation of risky choice tendency

The GD group (*n*=21) completed a behavioral session of the multi-step gambling task in the fMRI scanner (Figure 1a), and the resulting choice pattern was compared to the HC group data (*n*=29) obtained in the same period.^21^ The participants experienced a broad range of quota severity in a course of choice trials (Figure 1b), allowing us to examine the flexible change of risk attitude corresponding to the quota severity in an experimental session.

**Figure 2:**
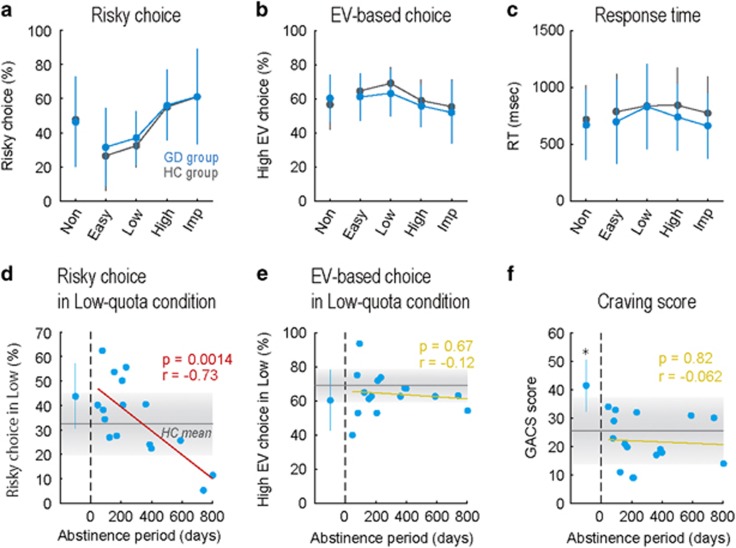
Behavioral results. (**a**) Risky choice probability in five quota conditions (Non, Easy, Low, High, Imp-quota conditions). Average data of GD group (*n*=21, cyan line, mean and SD) and HC group (*n*=29, gray line) are plotted. (**b**) Expected-value (EV)-based choice probability in five quota conditions. (**c**) Response time (RT) in five quota conditions. (**d**) Relationship between abstinence period (abscissa) and risky choice probability in low-quota condition (ordinate). Dots and fitted line configure data of under-treatment patients in GD group (*n*=16). Left-most plot with error bar indicates mean and SD of active gamblers in GD group (*n*=5). Horizontal gray line with shading indicates mean and SD of HC group data. (**e**) Relationship between abstinence and EV-based choice probability in low-quota condition. (**f**) Relationship between abstinence and self-reported craving level (GACS score). Asterisk indicates significant difference of GACS score between active gamblers and under-treatment patients or HC group (*P*<0.01, rank-sum test). GACS, Gambling Craving Scale; GD, gambling disorder; HC, healthy controls.

Both groups showed robust change of risky choice probability (Figure 2a) and EV-based choice probability (Figure 2b) depending on the quota condition. Two-way ANOVA (quota × group) revealed a main effect of quota (Non/Easy/Low/High/Imp) in risky choice probability (F_(4,240)_=19, *P*<0.001) and in EV-based choice probability (F_(4,240)_=6.2, *P*<0.001), but no main effect of group (HC/GD) in risky choice probability (F_(1,240)_=0.48, *P*=0.49) or in EV-based choice probability (F_(1,240)_=2.0, *P*=0.16). The response time (RT) was not statistically different by quota condition or by group (main effect of quota: F_(4,240)_=1.5, *P*=0.20; main effect of group: F_(1,240)_=3.0, *P*=0.087; Figure 2c). No significant quota × group interaction was observed in risky choice probability, in EV-based choice probability, or in RT (*P*>0.10). Thus, the GD group showed a statistically indistinguishable choice pattern from the HC group, in terms of average level.

### Condition-specific risky choice tendency of GD patients

In our GD population, the treatment duration was diverse; this could have influenced their choice patterns, and hence average analyses might have overlooked potential behavioral alteration in the GD group. In addition, as hypothesized in the Introduction, GD patients may be more likely to take unnecessary risky choice when they do not have to take the risk; this could be observed when they made decisions under low-quota condition in our paradigm. Therefore, we expected that GD patients with less abstinence show risky choice tendency in low-quota condition. Indeed, when we split the GD group into two subgroups based on abstinence duration, the shorter abstinence subgroup (*n*=11) showed risky choice tendency in low-quota condition, compared to the HC group (d.f.=39, *P*=0.033, rank-sum test).

To further investigate the relationship between abstinence and the risky choice tendency of the GD group, we performed a correlation analysis between abstinence period and risky choice probability for each quota condition. Prior computational simulation suggested optimal strategy in low/high-quota conditions: EV-based choice strategy for low-quota condition, and risky choice strategy for high-quota condition (Figures 1c and d, also see Supplementary Information). We thereby expected the GD group to violate this optimal pattern, such that patients with less abstinence show greater risky choice tendency in low-quota condition. As expected, we observed a strong, negative correlation between abstinence period and risky choice probability in low-quota condition in the under-treatment patients (*n*=16, *r*=−0.73, *P*=0.0014; Figure 2d). No significant correlation between abstinence and risky choice probability was observed in other quota conditions (*P*>0.10). In addition, EV-based choice probability was not correlated to the abstinence period under any quota conditions (*P*>0.10; Figure 2e). Thus, the GD group showed quota-condition-specific risky choice tendency (that is, failure of risk aversion in low-quota condition), and this reflected the treatment duration. Inflexibility of risk attitude in low/high-quota conditions suggests a deficit of state-dependent strategy optimization in the GD group.

### Self-reported craving level and trait impulsivity

We also analyzed the score of the Gamble Craving Scale (GACS)^30^ as a measure of the subjective craving level, and investigated the relationship between craving level and condition-specific risky choice tendency of the GD group. In this analysis, however, we found no correlation between the GACS score and risky choice probability in low-quota condition in the GD group (*n*=21, *r*=0.14, *P*=0.55). Moreover, unlike the risky choice probability in low-quota condition, the GACS score was high only in active gamblers, exhibiting a step-like pattern (Figure 2f). Active gamblers (*n*=5) showed significantly higher GACS score than under-treatment patients (d.f.=19, *P*=0.0056, rank-sum test) and the HC group (d.f.=32, *P*=0.0098, rank-sum test), and no correlation was found between abstinence period and GACS score in under-treatment patients (*n*=16, *r*=−0.062, *P*=0.82). Thus, the suboptimal risky choice tendency observed in the GD group was not directly accounted for by heightened craving level.

In addition, we analyzed the score of the BIS/BAS scale^31^ as a measure of trait impulsivity. Although the BIS/BAS score of the GD group was significantly higher than that of the HC group (d.f.=48, *P*=0.016, rank-sum test), it was not correlated to risky choice probability in low-quota condition (*n*=21, *r*=0.26, *P*=0.26) or to abstinence period (*n*=21, *r*=−0.20, *P*=0.38). Thus, trait impulsivity also did not account for suboptimal risky choice tendency in the GD group.

### Attenuation of quota-dependent neural activity in GD group

**Figure 3:**
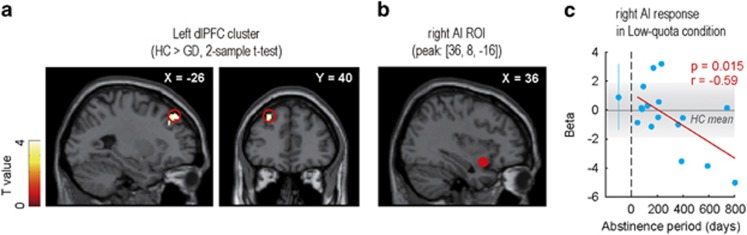
Activation analysis. (**a**) Result of two-sample *t*-test using quota severity contrast ([0 −2 −1 1 2]). Red open circles indicate left dlPFC cluster on a sagittal slice (left) and on a coronal slice (right). The peak coordinates were at [−26, 40, 44]. The threshold was set at *P*<0.05 with cluster-size correction. (**b**) Red-filled circle on a sagittal slice indicates right AI ROI (peak coordinates: [36, 8, –16]). (**c**) Relationship between abstinence period (abscissa) and right AI ROI response in low-quota condition (ordinate). Beta represents the regression coefficient of GLM regarding single contrast of low-quota condition ([0, 0, 1, 0, 0]). Schema of the figure is the same as Figures 2d–f. AI, anterior insula; dlPFC, dorsolateral prefrontal cortex; GLM, general linear model.

We next analyzed the fMRI data obtained during task execution. Because behavioral analyses highlighted the deficit of quota-dependent strategy modulation in the GD group, we assumed that neural encoding of quota severity would be attenuated in the GD group. In the previous study, we reported that the dlPFC, dorsal anterior cingulate cortex (dACC) and right anterior insula (AI) reflected the ‘quota severity' contrast (Imp>High>Low>Easy) in healthy participants.^21^ Therefore, we expected the GD group to show weakened encoding of quota severity in these areas. As expected, the two-sample *t*-test (HC>GD) revealed a significant cluster in the left dlPFC, suggesting diminished encoding of quota severity in the GD group (Figure 3a).

There was no significant cluster in dACC or right AI, but these areas might be involved in a more condition-specific role. Then, we additionally performed correlation analysis between abstinence period and ROI responses in low-quota condition for dACC and right AI ROIs (peak: dACC [2, –4, 48], right AI [36, 8, –16]). ROIs were set on the peak coordinates identified in one-sample *t*-test of the HC group data. The ROI response in low-quota condition was calculated for each participant by using the single contrast for low-quota condition ([0 0 1 0 0]). This analysis revealed a negative correlation between abstinence period and right AI response in low-quota condition in under-treatment patients (*n*=16, *r*=−0.59, *P*=0.015; Figures 3b and c), which resembled the behavioral correlation pattern in Figure 2d. This relationship was not observed when we set ROI on dACC (*n*=16, *r*=0.29, *P*=0.27).

### Alteration of quota-dependent functional connectivity pattern in GD group

In the previous study, we employed the gPPI method^32^ and reported the neural correlates of state-dependent strategy optimization. The functional connectivity pattern between the left dlPFC and dmPFC encoded ‘strategy optimization' contrast (Low/High>Non/Imp), and the strength of dlPFC–dmPFC connectivity reflected the task performance (that is, negative correlation between dlPFC–dmPFC connectivity and risky choice probability in low-quota condition) in the HC group.^21^ Therefore, we performed a gPPI analysis with left dlPFC seed (peak: [-26, 40, 44], Figure 3a), and compared the functional connectivity patterns of the two groups.

**Figure 4:**
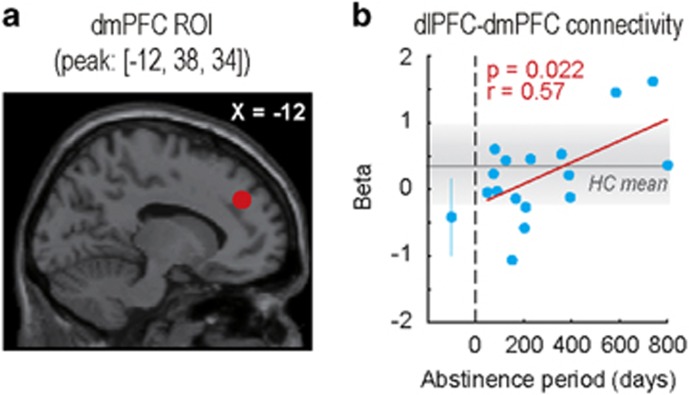
Functional connectivity analysis with left dlPFC seed. (**a**) Red-filled circle on a sagittal slice indicates dmPFC ROI (peak coordinates: [−12, 38, 34]). (**b**) Relationship between abstinence period (abscissa) and dmPFC ROI response (ordinate). Beta represents the regression coefficient of GLM regarding ‘strategy optimization' contrast ([−1 0 1 1 −1]). Schema of the figure is the same as Figures 2d–f. dlPFC, dorsolateral prefrontal cortex; dmPFC, dorsomedial prefrontal cortex; GLM, general linear model.

In the two-sample *t*-test (HC>GD), we found no significant cluster reflecting a difference of encoding of state-dependent strategy optimization. However, taking into account the fact that dlPFC–dmPFC functional connectivity was negatively correlated to the risky choice probability in low-quota condition in the HC group, it might also reflect the abstinence duration and thus appear indistinguishable by two-sample *t*-test. We then investigated the relationship between abstinence period and dlPFC–dmPFC functional connectivity by ROI analysis with dmPFC ROI (peak: [−12, 38, 34]). The ROI was set on the peak coordinates of the dmPFC cluster identified by one-sample *t*-test of the HC group data (Figure 4a). The ROI response was calculated for each participant by using strategy-optimization contrast ([−1 0 1 1 −1]). We found that the dmPFC ROI response was positively correlated to the abstinence period in the GD group (*n*=16, *r*=0.57, *P*=0.022; Figure 4b), suggesting a relationship between the dlPFC–dmPFC functional connectivity pattern and the degree of recovery. Moreover, the dmPFC ROI response was negatively and marginally correlated to the risky choice probability in low-quota condition in the GD group (*n*=21, *r*=−0.42, *P*=0.061), suggesting a relationship between dlPFC–dmPFC functional connectivity pattern and risk aversion in low-quota condition of the GD group. Thus, fMRI analyses highlighted that not only the activation pattern but also the functional connectivity pattern was altered in GD patients.

## Discussion

Although a wealth of studies have focused on the individual risk preference of GD patients, the role of state-dependent strategy optimization in GD has never been investigated. In the present study, by manipulating the contextual state (quota severity) in a multi-step gambling task (Figure 1a), we found a quota condition-specific suboptimal choice tendency in the GD group. The risky choice probability of GD patients was high in low-quota condition (Figure 2d), in which the EV-based choice was defined as the optimal strategy, suggesting a failure of optimizing risk attitude when they should avoid risk. Because this quota-condition-specific risky choice tendency was not correlated to the self-reported craving level (GACS score; Figure 2f) or to trait impulsivity (BIS/BAS score), it was not directly accounted for by general up-shifting of the subjective craving level or trait impulsivity. Thus, for we believe the first time, we found a deficit of state-dependent strategy optimization in GD patients.

Deficit of state-dependent strategy optimization may account for part of GD's risk-taking. In a daily-life situation, low-quota condition could reflect small monetary loss. Risky tendency for small monetary loss can induce unnecessary gambling, and repetition of such gambling can lead to financial hardship in GD subjects. Hence, in addition to the abnormality of individual risk preference, deficit of state-dependent strategy optimization may play a crucial role in the GD mechanism.

Functional MRI analysis revealed diminished encoding of quota severity in dlPFC in the GD group (Figure 3a). The dlPFC has been emphasized for its role in cognitive flexibility,^22, 23^ and it could have mediated flexible strategy optimization depending on the quota condition in the current paradigm. Hence, the weaker activation pattern of dlPFC in the GD group may reflect impairment of cognitive flexibility. In fact, some studies have reported less cognitive flexibility in GD patients,^33, 34^ suggesting impairment of cognitive flexibility underlying the deficit of state-dependent strategy optimization in GD.

In addition, we found negative correlation between abstinence period and right AI ROI response in low-quota condition (Figures 3b and c), suggesting a relationship between right AI activity and the degree of recovery. Past studies reported that human subjects with insula damage frequently experienced natural quitting of addictive cigarette smoking^35, 36^ and expressed a weaker level of gamble-related cognitive biases (near-miss effect and gambler's fallacy),^37, 38^ highlighting a possible causal role of AI in GD. Considering that right AI encoded quota severity in the HC group,^21^ reduction of right AI response might have reduced the perceived quota and led to conservative choices in patients with long abstinence.

In the functional connectivity analysis by gPPI method, we found a positive correlation between the abstinence period and encoding of state-dependent strategy optimization in dlPFC–dmPFC functional connectivity (Figure 4b). This encoding pattern further reflected the task performance in the GD group, that is, optimal risk aversion in low-quota condition, suggesting a crucial role of dlPFC–dmPFC functional connectivity for recovery. Because dmPFC has been implicated in prospective thinking,^39, 40, 41^ dlPFC–dmPFC could play a pivotal role in prospective goal-setting. Indeed, past studies reported poor performance of GD patients in intertemporal choice task, in which GD patients preferred a smaller-and-sooner option over a larger-and-later option and failed to maximize the over-trial profit.^8, 42^ Therefore, attenuation of the dlPFC–dmPFC functional connectivity pattern in GD patients could reflect a deficit in prospective goal-setting and subsequent strategy optimization.

The current study has several limitations. First, although GD patients showed blunted brain signals for tracking quota, they did not differ from the HC group in terms of overall risky choice probability. Our behavioral measures might not have been sensitive enough to reflect the attenuation of brain signals. Second, this is a cross-sectional study, and therefore the relationship between brain activity and abstinence period remains to be correlational. Longitudinal studies are highly recommended to test the causal relationship between them.

In conclusion, the present study sheds light on the novel mechanism of GD's abnormal risk-taking. Deficit of state-dependent strategy optimization was reflected in behavioral and neuronal patterns of GD patients and potentially accounted for part of GD's abnormal risk-taking. It is also noteworthy that the important neural circuits for state-dependent strategy optimization were distributed outside of reward-related areas (dlPFC, dmPFC and AI) that have received less attention in past literatures concerning GD. We believe that the present study will contribute to a better understanding of GD and may be useful for the development of new therapies.
