# Supplementary material for: Deficit of state-dependent risk attitude modulation in gambling disorder
Source: Transl Psychiatry. 2017 Apr 4;7(4):e1085–. doi: 10.1038/tp.2017.55 (PMC5416696; doi:10.1038/tp.2017.55)
Supplement: Supplementary Information [file tp201755x1.doc]

**Supplementary Information**

**Supplementary Methods**

***Participants***

Twenty-four male GD patients participated in the present study, and 21 GD patients without comorbidity (GD group; Age: 34.7±8.82 years; 20 right-handed) and 29 age-matched healthy participants (HC group; Age: 30.9±10.4 years; 27 right-handed) were analyzed. No HC group members had a history of neuronal/psychiatric disorders. Intelligence Quotient (IQ) was also matched between the two groups (Japanese Adult Reading Test; GD group: 107±7.08; HC group: 104±8.54).

In the GD group, 5 patients were active gamblers with no treatment history. The remaining under-treatment patients (n = 16) were recruited from a residential treatment facility (Serenity Park Japan, Nara, Japan), where patients underwent psychological therapy (twelve-step program) and were maintaining abstinence (222±241 days). All of the GD group played a slot machine or a variant of a slot machine, “Pachinko”.

***Behavioral Data Analysis***

Behavioral analyses and following fMRI analyses were performed with MATLAB R2010a (MathWorks, Natick, MA).

Five quota conditions were defined based on a prior computational simulation. In the simulation, an agent representing expected-value-based choice strategy (EV model) and that representing risky choice strategy (RISKY model) performed a multi-step gambling task with identical settings to human participants. The performance of each agent was evaluated as the proportion of block clear (solving a block quota) in a given session (10,000 blocks). Although the EV model was the rational agent to maximize average earnings (Figure 1c, left), the RISKY model beat the EV model when they competed on an extremely severe quota, because the RISKY model yielded the largest units in a block (Figure 1c, righ**t**). The relative dominance of the RISKY model over the EV model was assessed as CP-IDX, which represented the relative block-clear probability of RISKY model compared to EV model.

$CP\text{‐}IDX=\frac{\left\{CP\left(RISKY\right)-CP(RAND)\right\}-\left\{CP\left(EV\right)-CP\left(RAND\right)\right\}}{\left\{CP\left(RISKY\right)-CP(RAND)\right\}+\left\{CP\left(EV\right)-CP\left(RAND\right)\right\}}$,

where CP(RISKY), CP(EV), and CP(RAND) represented the block-clear probability of the RISKY model, EV model, and random choice, respectively. Importantly, the valence of CP-IDX was reversed at an indifferent point (310 units/trial), indicating that the optimal strategy for block clear was switched at this point (Figure 1d). In addition, random choice yielded 131 units/trial on average.

Based on the result of simulation, five quota conditions were defined. ‘Non-quota condition’ (0 units/trial) was comprised of the trials after block clear was secured (for instance, if a participant achieved block quota at the 17th trial, the remaining 3 trials in the block were analyzed as Non-quota condition). ‘Easy-quota condition’ (0-131 units/trial) was set below the average score of random choices, and therefore no strategic choice was required. In ‘Low-quota condition’ (131-310 units/trial), the EV-based choice strategy was the optimal strategy to maximize the block-clear probability. In ‘High-quota condition’ (310-600 units/trial), the risky choice strategy was the optimal strategy to maximize the block-clear probability. ‘Imp-quota (impossible quota) condition’ (> 600 units/trial) was set above maximum payoff for a trial so that no strategy would clear this condition.

***fMRI Data Acquisition***

The scanning session was conducted at Kokoro Research Center, Kyoto University, Japan. A 3T scanner equipped with a 32-channel head coil (Verio, Siemens, Erlangen, Germany) was used. Functional images in a T2*-weighted echo-planner imaging (EPI) sequence (TR = 2500 ms; TE = 5.19 and 7.65 msec; flip angle = 60°; FOV = 192×192 mm; matrix = 64×64; voxel size = 3×3×3 mm), and anatomical images in a T1-weighted MPRAGE sequence with identical scanning parameters to the EPI sequence were obtained. The first two volumes were discarded to allow for signal stabilization.

***fMRI Activation Analyses***

Statistical Parametric Mapping (SPM8; Wellcome Department of Imaging Neuroscience, University College London, UK) run on MATLAB was used for fMRI data analyses. The images underwent realignment, slice-timing correction, normalization to the template, and smoothing with an 8-mm isotropic Gaussian kernel.

In the first-level analysis, five regressors corresponding to the stimulus-onset time of the five quota conditions (Non, Easy, Low, High, Imp) were implemented in the general linear-regression model (GLM) with motion parameters. Then ‘quota-severity’ contrast [0 -2 -1 1 2], representing the strictness of quota severity (Imp > High > Low > Easy), was tested for each participant. In group-level analysis, encodings of quota-severity contrast in the GD and HC groups were compared by two-sample t-test. We used a combination of height threshold uncorrected P < 0.001 and cluster-size threshold 69 voxels (corrected for multiple comparisons, P < 0.05). For additional region-of-interest (ROI) analysis, ROIs (6-mm sphere) were set on the peak coordinates of clusters (dACC [2, -4, 48], right AI [36, 8, -16]) that were identified in one-sample t-test of HC group data (quota-severity contrast) with cluster-forming threshold P < 0.001.

***fMRI Functional-Connectivity Analysis***

The generalized form of the context-dependent psychophysiological interaction (gPPI) method was employed for functional-connectivity analysis.

In the first-level analysis, five PPI regressors were constructed with the time course of seed region and onset-time of each quota condition, and implemented in a single GLM with physiological regressor and psychological regressors. We set the seed (6-mm sphere) on the peak coordinates of the left dlPFC cluster ([-26, 40, 44]), which were identified in activation analysis. Then the ‘strategy-optimization’ contrast [-1 0 1 1 -1] (Low/High > Non/Imp) was tested to search for neural correlates of the utilization of state-dependent strategy optimization for each participant. In group-level analysis, encodings of strategy-optimization contrast in the GD and HC groups were compared by two-sample t-test. We used a combination of height threshold uncorrected P < 0.01 and cluster-size threshold 64 voxels (corrected for multiple comparisons, P < 0.05). For additional ROI analysis, ROI (6-mm sphere) was set on the peak coordinates of the dorso-medial prefrontal cortex (dmPFC) cluster ([-12, 38, 34]), which was identified in one-sample t-test of HC group data (strategy-optimization contrast) with the cluster-forming threshold P < 0.01.
